# Going wild: prioritizing experimental models of rodent-borne viruses to decode zoonotic spillover and pandemic risk

**DOI:** 10.1128/jvi.01751-25

**Published:** 2026-05-27

**Authors:** Rebekah Honce, Jason Botten

**Affiliations:** 1 Department of Animal and Veterinary Sciences, College of Agriculture and Life Sciences, University of Vermont 173139, Burlington, Vermont, USA; 2 Division of Pulmonary and Critical Care Medicine, Department of Medicine, Robert Larner, M.D. College of Medicine, University of Vermont 12352, Burlington, Vermont, USA; 3 Department of Microbiology and Molecular Genetics, Robert Larner, M.D. College of Medicine, University of Vermont 12352, Burlington, Vermont, USA; New York University Department of Microbiology, New York, New York, USA

**Keywords:** disease ecology, arenavirus, hantavirus, zoonotic infections, animal models

## Abstract

Rodent-borne viruses pose threats to global health because of their capacity for zoonoses with the potential to cause outbreaks of high morbidity and mortality. Gaining a deeper understanding of how these viruses persist in their reservoir hosts and spillover into human populations requires an integrated approach blending both laboratory- and field-based research. Here, we review our current understanding of rodent-borne virus dynamics—primarily mammarenaviruses and orthohantaviruses—within their natural rodent reservoirs. We highlight key insights gained from experimental infection models seeking to replicate authentic host-pathogen interactions under controlled conditions. We argue that expanding and refining experimental models, particularly those that simulate natural reservoir conditions, is crucial to identifying the host and viral factors facilitating viral persistence in nature, and transmission to human populations. We propose a framework for future research that prioritizes hypothesis-driven research in model systems that are both natural and translatable to bridge these knowledge gaps.

## INTRODUCTION

Rodents have been a cornerstone of medical research since the 17th century, with their use formalized alongside the development of fancy mice into familiar inbred mouse strains in the early 1900s (1). As model organisms, rodents are invaluable in the study of human disease. However, *Mus musculus* is just one member of the diverse Order Rodentia, which comprises approximately 43% of all mammalian species. This incredible species richness, combined with their often synanthropic life history, makes rodents a high-risk group for zoonotic spillovers (2–5). While *Mus* is only a source for fewer than two dozen human pathogens and a primary reservoir of just one medically important virus (2, 6), Rodentia species serve as primary reservoirs for a wide range of high-priority threats, including viruses of pandemic and bioterror potential (Table 1).

**TABLE 1:** Primary reservoirs of medically important rodent-borne viruses^*g*^

Virus		Reservoir	
Family	Species^*a*^	Family	Species
Arenaviridae	Junín virus (*Mammarenavirus juninense*)	Cricetidae	Drylands vesper mouse (*Calomys musculinus*) (7, 8); Crafty vesper mouse (*Calomys callidus*) (9); Azara’s grass mouse (*Akodon azarae*) (10), Molina’s grass mouse (*Akodon molinae*) (7)
	Machupo virus (*Mammarenavirus machupoense*)	Cricetidae	Large vesper mouse (*Calomys callosus*) (11)
	Guanarito virus (*Mammarenavirus guanaritoense*)	Cricetidae	Short-tailed cane mouse (*Zygodontomys breviacauda*) (12); Hispid cotton rat (*Sigmodon hispidus*) (13)
	Chapare virus (*Mammarenavirus chapareense*)	Unknown	Unknown
	Sabia virus (*Mammarenavirus brazilense*)	Unknown	Unknown
	Lymphocytic choriomeningitis virus (*Mammarenavirus choriomeningitis*)	Muridae	House mouse (*Mus musculus*) (14)
	Lassa virus (*Mammarenavirus lassaense*)	Muridae	Natal multimammate mouse (*Mastomys natalensis*) (15)
	Lujo virus (*Mammarenavirus lujoense*)	Unknown	Unknown
Hantaviridae	Sin Nombre virus (*Orthohantavirus sinnombreense*)^*b*^	Cricetidae	Deer mouse (*Peromyscus maniculatus*) (16, 17); Cloudland deer mouse (*Peromyscus maniculatus nubiterrae*) (18); White-footed mouse (*Peromyscus leucopus*) (19)
	Choclo virus (*Orthohantavirus chocloense*)	Cricetidae	Northern pygmy rice rat (*Oligoryzomys fulvescens*) (20)
	Bayou virus (*Orthohantavirus bayoui*)	Cricetidae	Marsh rice rat (*Oryzomys palustris*) (21)
	Black Creek Canal virus (*Orthohantavirus nigrorivense*)	Cricetidae	Hispid cotton rat (*Sigmodon hispidus*) (22)
	Andes virus (*Orthohantavirus andesense*)	Cricetidae	Long-tailed rice rat (*Oligoryzomys longicaudatus*) (23)
	Rio Mamore virus (*Orthohantavirus mamorense*)	Cricetidae	Small eared pygmy rice rat (*Oligoryzomys microtis*) (24)
	Puumala virus (*Orthohantavirus puumalaense*)	Cricetidae	Bank vole (*Myodes glareolus*) (25)
	Dobrava virus (*Orthohantavirus dobravaense*)^*c*^	Muridae	Striped field mouse (*Apodemus agrarius*) (26, 27); Yellow-necked mouse (*Apodemus flavicollis*) (28); Black Sea field mouse (*Apodemus ponticus*) (29, 30)
	Seoul virus (*Orthohantavirus seoulense*)	Muridae	Brown rat (*Rattus norvegicus*) (31)
	Hantaan virus (*Orthohantavirus hantanense*)^*d*^	Muridae	Striped field mouse (*Apodemus agrarius*) (32); Korean field mouse (*Apodemus peninsulae*) (33)
Flaviviridae	Omsk hemorrhagic fever virus (*Orthoflavivirus omskense*)^*e*^	Cricetidae	Common muskrat (*Ondatra zibethicus*) (34)
Poxviridae	Mpox virus (*Orthopoxvirus monkeypox*)	Sciuridae	African rope squirrels (*Funisciurus anerythrus*)^*f*^ (35, 36); Prairie dog (*Cynomys* sp.) (37)

^*a*^ ICTV species and name designations (38, 39).

^*b*^ Encompasses Sin Nombre virus, Monongahela virus, and New York virus.

^*c*^ Encompasses Dobrava virus, Saaremaa virus, and Sochi virus.

^*d*^ Encompasses Hantaan virus and Amur virus.

^*e*^ Carried by ticks, but infection via contact with the amplifying rodent host has been reported.

^*f*^ Earliest suggested reservoir host, although species across at least 20 mammalian families are implicated as reservoirs or amplifying hosts (40).

^*g*^ Additional information is combined from previous studies (41–43) and Fields Virology Chapters *Bunyavirales; *Orthohantaviruses, Orthonairovirus, Orthobunyavirus and Phlebovirus; and *Arenaviridae *(44).

Rodent-borne viruses—such as *Mammarenavirus* and *Orthohantavirus* genera—were traditionally thought to follow a “one virus, one host” model, with each maintained in a single rodent species (45, 46). However, recent surveillance suggests that these viruses circulate either consistently or transiently across multiple hosts (41, 47). For many rodent-borne zoonotic diseases, long-term co-evolution between the virus and host has resulted in persistent, asymptomatic infections—contrasting sharply with the acute, often severe disease seen in incidental human hosts (48, 49). However, the molecular mechanisms driving this host-virus equilibrium remain poorly understood. Given the predicted increase in zoonoses, it is crucial to study the natural history of rodent-borne viruses within their primary hosts to better understand their sylvatic cycles and factors driving their emergence. This knowledge is essential for developing effective prevention and control strategies to mitigate future outbreaks.

However, this research poses technical and intellectual challenges. Here, we examine how medically important rodent-borne viruses are maintained within their natural reservoirs, emphasizing empirical studies conducted in authentic host species. We compare these findings to other high-priority virus-host systems and discuss the value and obstacles of studying zoonotic viruses in their native hosts. While many studies have investigated arenavirus and hantavirus infections using laboratory strains of *Mus* and *Rattus* as models for human disease (50–52), fewer have employed authentic host-virus systems to explicitly study reservoir biology. Consequently, our understanding of the natural dynamics of these viruses remains incomplete.

## EXPERIMENTAL INFECTIONS OF RODENT RESERVOIR SPECIES

Surveillance and field studies remain indispensable for rodent-borne virus detection, discovering reservoir species, mapping prevalence and geographic range, and characterizing the ecological context of infection across space and time (53, 54). However, drawing mechanistic conclusions from these data can be challenging due to spatiotemporal limits on sampling and methodological heterogeneity—including differences in tissue collection, diagnostic platforms (molecular, serological, or infectious agent-based), sampling timing relative to infection, and the depth of associated metadata (55, 56). While reporting standards have enhanced data quality and comparability across studies (57–59), a critical gap remains in linking these patterns to mechanisms underlying pathogenesis, persistence, and transmission. To address this, we focus here on experimental infection models in authentic reservoir hosts as a complementary bridge between field ecology and conventional laboratory models, allowing for controlled tests of viral biology that are difficult to study in nature and providing key baseline insights into the natural history of infection occurring in reservoir animals. We summarize the insights gained to date for key virus-host pairs and outline practical considerations for developing, interpreting, and advancing these systems in future work.

### Arenaviridae

**Fig 1:**
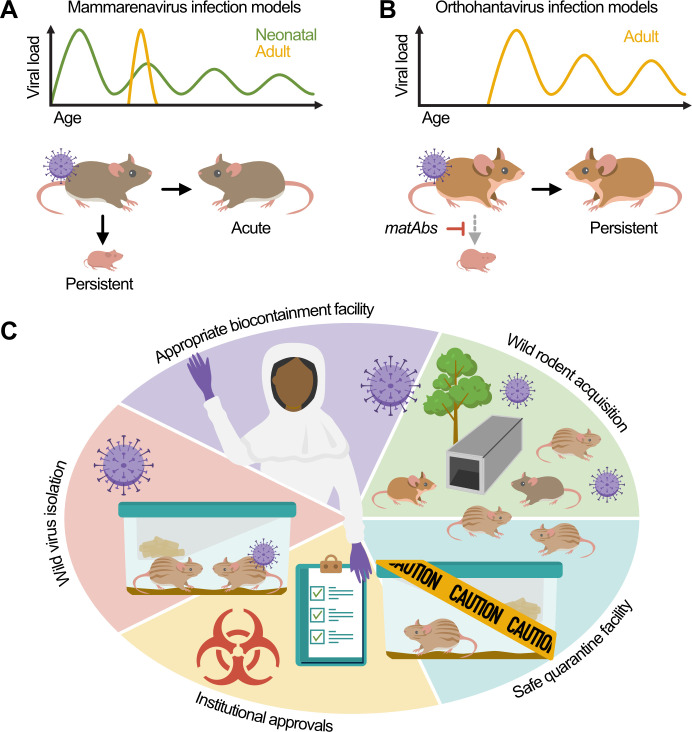
Generalized experimental infection models of rodent-borne viruses. (**A**) Unlike the acute, self-limiting infections typically seen in adult rodents, mammarenavirus infection in neonates often leads to persistent viral carriage with minimal clinical symptoms. This persistence facilitates vertical transmission in experimental infection systems that mimic the hypothesized route in nature, whereby infected adult female mice transmit the virus to their offspring *in utero*. Horizontal transmission, by contrast, generally results in an acute infection and viral clearance within 1–2 weeks. (**B**) In the case of orthohantaviruses, vertical transmission is generally thought to be blocked by maternally derived neutralizing antibodies. Consequently, horizontal transmission between adult rodents serves as the predominant route of viral spread in nature and can be assessed in the laboratory setting. (**C**) The path to success in establishing wild-caught rodent colonies for experimental research requires (i) the ability to capture sufficient numbers of wild rodents, (ii) a suitable safe location for quarantine, (iii) institutional approval to house and breed wild rodents in standard laboratory environments, (iv) isolating the targeting virus from the rodent population from which the breeders were obtained, and (v) availability of high containment to safely conduct experimental infections. Artistic representations were designed by Freepik (rodent species, virus, tree) and studiogstock (clipboard) and are used under a CC BY 4.0 license or were designed by the authors.

Infection outcomes in mammarenavirus models vary by host age, inoculation route, dose, and viral strain. Neonatal infection often not only leads to persistent carriage and vertical transmission but also stunts growth, reduces lifespan, and lowers reproductive success (60–62). In contrast, immunocompetent adults typically clear acute infections within a week (63). However, this dogma stems from studies with laboratory-adapted hosts and viral isolates and thus may not reflect their natural dynamics (64–66). Experimental infection models have been well-developed for three systems: lymphocytic choriomeningitis (LCMV)*-Mus,* Lassa (LASV)*-Mastomys,* and Junín (JUNV)*-Calomys* (Fig. 1A).

#### Lymphocytic choriomeningitis virus and *Mus*

Despite its foundational role in immunology, LCMV remains relatively understudied in a virologic context. Its natural reservoir, *Mus musculus*, has long served as a cornerstone of biomedical research. However, reliance on inbred mouse strains to study common LCMV strains—also highly laboratory-adapted—introduces significant limitations. Increasingly criticized for their poor translatability to humans, inbred mouse strains should also not be seen as representations of wild *Mus* (67–71). This model may not accurately reflect LCMV dynamics in wild populations of *Mus* or its broader viral ecology. With those caveats, LCMV infection outcome in laboratory mice varies depending on host genetics, age, and immunocompetence as well as viral strain, dose, and route of inoculation (72, 73).

Immunocompetent adult models featuring Armstrong-like strains are typically self-limiting acute infections but have found use as an immunological tool (74–76). Instead, to better model the sylvatic cycle of LCMV, two approaches are common: intraperitoneal infection of neonates and congenital transmission via persistently infected breeding pairs (72). These models hinge on infection prior to the development of a mature adaptive immune response. Early-in-life infection is thought to lead to negative selection of virus-specific lymphocytes, allowing viral persistence (77). During persistence, LCMV antigen, genome, and infectious virus are detectable across many tissues and are excreted in saliva, urine, blood, and feces (64, 78–82). High levels of shedding enable horizontal transmission (83), a route not expected to be a major driver of viral maintenance, as it does not trigger persistency in adults; however, it can lead to infection of developing embryos if maternal infection occurs early in pregnancy (83, 84). Instead, vertical transmission is the hypothesized major natural route with infection of the ovaries, ova, and/or placenta, facilitating transmission to the embryo (64, 84–86). During persistent infection in these models, there are minimal clinical signs of disease (64, 80), although long-term survival and reproductive outputs may be impaired (79, 81, 86, 87). This is especially apparent for directly infected neonatal mice that often exhibit runting and reduced fecundity, but these traits disappear in subsequent generations of persistently infected mice (64, 80, 88). This transient phenotype resembles findings in JUNV*-Calomys*, although its mechanistic basis is still unclear.

Commonly used LCMV strains differ in tropism, replication, and immune stimulation in laboratory mice (72, 89). In contrast to the persistent models utilizing Armstrong outlined above, chronic infections can be established in adult immunocompetent mice using strains such as Docile, WE, or Clone 13. In studies comparing the slow-growing LCMV-Docile with WE, attenuated viral replication early in infection may facilitate persistence through reducing immune activation; however, studies comparing Armstrong and Clone 13—which differ by only two amino acids—showed that the increased replicative capacity of Clone 13 in antigen-presenting cells resulted in immunosuppression and persistence (61, 90, 91). While Clone 13 is a prototypic model, this chronic infection in adult *Mus* differs from congenitally derived persistence in the wild and should not, along with WE, be considered a direct analog of natural infection (80, 88).

However, these chronic models provide insight into LCMV-mediated immune modulation co-occurring with the largely asymptomatic persistent state (92). Persistent antigen stimulation leads to T cell exhaustion, characterized by the loss of CD4+-mediated effector functions and upregulation of inhibitory PD-1 and LAG-3 signaling (93–96). This exhaustion results in the partial failure of T cell-mediated viral clearance but prevents host-damaging viral replication. Alterations to innate and B cell-derived cytokines can also alter the control of infection; their roles in the immunological adaptations during LCMV persistency in more naturalistic models are to be determined (97–99). Although virus-specific antibodies are made, heavy glycan shielding of the viral glycoprotein impairs the formation of neutralizing antibodies (100), which are only weakly detected late in infection (101–103). This contrasts with the strong neutralizing antibody response to orthohantavirus infection and supports the role of vertical transmission in LCMV maintenance. Of the limited number of LCMV genomes deposited in the Bacterial and Viral Bioinformatics Resource Center as of 2025 (*n* = 608) (104), less than 40% were derived from non-human or laboratory sources, only approximately 15% are complete genomes, and even fewer have been empirically studied in animal models. One such strain, LCMV-MN, can establish a persistent infection phenotype intermediate between acute and chronic laboratory strains when delivered to adult immunocompetent mice via intravenous injection, with transient systemic viremia but sustained tissue infection, reminiscent of what is thought to occur in naturally infected wild *Mus* (96). This model generates abundant progenitor exhausted CD8+ T cells, suggesting that T cell exhaustion may contribute to the maintenance of persistent LCMV infection as seen in previous studies (93–96). Increased scrutiny of LCMV strains to identify conserved viral factors that drive either pathogenesis or asymptomatic persistence will aid in defining how LCMV is generationally maintained in wild *Mus*.

#### Lassa virus and *Mastomys*

Like LCMV, experimental LASV infection of its principal host, *M. natalensis*, yields age-dependent infection clarified by the establishment of a wild-caught breeding colony (105–107). In experimentally infected adult *Mastomys,* LASV causes little clinical disease or pathology despite high levels of viremia and viral shedding, contrasting with the infection of non-reservoir hosts and development of Lassa hemorrhagic fever in humans (108). Peak LASV RNA levels are detected within 2 weeks of inoculation, with clearance generally by 4–6 weeks (106). In contrast, infection of neonatal *M. natalensis* within the first 2 weeks of life results in persistent viral carriage across multiple tissues, accompanied by no effect on growth or histological indications of disease (105). LASV RNA could be detected for at least 40 weeks (105, 109, 110).

The establishment of laboratory-reared, persistently infected *M. natalensis* adults has enabled the study of LASV transmission and finer insight into cellular responses to infection. Offspring of persistently infected females also displayed high viremia, but intriguingly, antibody levels declined with each subsequent generation during long-term breeding (105). Like LCMV, vertical transmission of LASV ensuing from mating of persistently infected males to naive females was possible, but not efficient. The resulting acute infection in dams led to transmission to less than one-quarter of the resulting offspring (105, 109). Intravaginal inoculation of female and intracutaneous inoculation of male *Mastomys* can lead to systemic infection, suggesting that sexual transmission and subsequent infection of fetal tissue may be possible (109). This is unlike studies with New World mammarenaviruses but similar to epizoological studies in laboratory *Mus* infected with LCMV (64), suggesting that overall, New World viral-host pairs may rely more heavily on horizontal transmission post-birth or between adults than Old World mammarenaviruses (60). Neonatally infected pups can also transmit virus to uninoculated but co-housed neonates and adult parents; however, persistency was again only established in neonatal contacts (105).

#### Junín virus and *Calomys*

Junín virus (JUNV) is maintained in cricetid rodents, primarily the reservoir *Calomys musculinus*, with other cricetids such as *Calomys laucha* and *Akodon molinae* implicated as alternative hosts. Historically, *Calomys* have been used as laboratory models since the 1960s (111–113). *C. musculinus* neonates intranasally infected with wild-type JUNV Cba AN 9446 strain develop persistent infection but show marked pre-weaning reductions in weight gain and increased post-weaning mortality (60). In contrast, intraperitoneal infection with the attenuated XJCl13 strain causes >50% mortality preceded by ataxia, tremor, and severe encephalitis (114, 115). Intriguingly, JUNV fails to establish persistency in neonatal *C. laucha* but can establish persistency in *A. molinae*, although in the latter instance chronic viremia is accompanied by neuropathology and high mortality (116, 117). This suggests that there may be a viral strain, inoculation route, host species, and/or other variable(s) contributing to disparate infection outcomes as with LCMV in *Mus*. Adults can also become persistently infected, although only roughly half of experimentally infected adult *C. musculinus* develop chronic viremia and shed infectious virus (60, 62, 118). Further studies on this age-dependent phenomenon have used *ex vivo* models, with circulating peripheral blood mononuclear cells (PBMCs) isolated from adult *Calomys* failing to support JUNV replication, while neonatal cells do (119, 120); however, no clear mechanism is known.

Surviving *C. musculinus* adults infected as neonates tend to show cyclical viremia and widespread viral dissemination across tissues, including brain and spleen (60, 114). As antigenically distinct JUNV variants arise throughout persistency when compared to virus isolated during the acute stage, the intra-host population may diversify due to production of neutralizing antibodies (121–123). Depletion of thymocytes after inoculation with JUNV immediately after birth reduced clinical disease and increased survival from 10% to 50% (115). This enhanced survival was not associated with decreased viral load in the central nervous system but did mitigate JUNV-induced brain damage (115), suggesting that immunopathology may contribute to encephalitic disease in JUNV-infected neonates. This is reminiscent of T cell-driven pathology observed in LCMV-infected immunocompetent laboratory *Mus* and *Rattus* (124, 125).

Although dam-to-pup transmission during gestation or shortly after is widely considered the primary mechanism for JUNV maintenance in nature, some evidence challenges the sufficiency of vertical transmission alone (126, 127). Neonatally infected *C. musculinus* display a marked reduction in fertility and correspondingly poor vertical transmission compared to adult-infected animals (60, 127); however, similar findings with LCMV suggest that a multigenerational transmission model, which occurs naturally in the wild, is required to offset the observed fitness costs (64). Viral transmission *in utero* through direct infection of the fetus has not been seen. Postnatal transmission from a viremic dam to her offspring is substantial, with approximately 50% of pups exhibiting viremia at weaning (127). In contrast, surveillance studies support the role of horizontal transmission in JUNV ecology through observations of increased seropositivity in older adult males with visible scarring indicative of increased aggressive contacts, similar to orthohantaviruses (7, 128, 129). However, no empirical studies to our knowledge have assessed JUNV transmission in a controlled infection setting. Overall, the mechanism(s) by which JUNV and other New World mammarenaviruses are maintained in reservoir rodents in nature remains an open question.

#### Other mammarenaviruses

Like other New World mammarenaviruses, Machupo (MACV) and Latino (LATV) virus inoculation of neonatal *C. callosus* and Guanarito (GTOV) virus in neonatal and weanling *Z. brevicauda* results in persistency (62, 130–132). With MACV, viral antigens are detectable by immunofluorescence across multiple tissues and infectious virus present in blood and urine for at least 20 weeks (130, 131). In contrast, long-term LATV infection yields no detectable viremia (62). Adult survivors of neonatal MACV infection diverge into two patterns: Type A (“immunotolerant”) shows persistent viremia, little or no neutralizing antibody, anemia, and splenomegaly, whereas Type B (“immunocompetent”) clears the infection, likely through the generation of neutralizing antibodies (62, 130, 131). The basis for these divergent outcomes is unknown, but Type A is associated with a lower inoculation dose and during inbreeding of persistently infected colonies, implicating immune exhaustion or host genetic factors (62). The immunogenetic link is supported by surveillance studies, as while both viruses circulate in Bolivia, they occupy distinct geographic ranges, and animals from LATV-endemic areas are less susceptible to MACV infection than those from MACV-endemic areas (116). These differences in disease presentation extend to vertical transmission. Type A animals have reduced reproductive output despite normal sexual development, with persistently infected females producing only 5% of expected offspring, while persistently infected males sire smaller litters even when mated with naive females (62). This fitness cost was linked to fertilization and implantation failures, alongside MACV-driven pathology in embryonic tissue (62). Adults with a Type B infection pattern show no reproductive deficits (62). Finally, as seen with JUNV infection of *C. musculinus,* adult *C. callosus* inoculated with MACV showed no clinical disease, but only a fraction remained viremic after 4–6 months, possibly due to high levels of neutralizing antibodies (130, 131). For GTOV, persistency can also be established in adults, but with less frequency than infection of neonates and weanlings (12, 132). In all cases, lifelong GTOV infection is accompanied by persistent viremia and shedding of infectious virus in oropharyngeal secretions and urine (132). Fewer studies examine Old World systems. Neonatal *Mastomys* infected with the nonpathogenic Morogoro virus (MORV) show only transient growth suppression, with MORV RNA peaking at day 10 and detectable by day 40 in urine, saliva, and feces (133). Because the primary reservoirs of many New and Old World mammarenaviruses remain incompletely defined and undiscovered mammarenaviruses may exist, our understanding of their natural history remains limited.

#### Future directions

Despite decades of work in laboratory systems, major gaps remain in understanding how mammarenaviruses are maintained in natural rodent populations. Future work should prioritize developing and reviving experimental infection models, such as the *Calomys*-JUNV system, as authentic reservoir hosts and minimally passaged viral isolates may better reflect natural host-virus interactions. Such models will be critical for resolving why closely related rodent species differ in their capacity to sustain persistent infection, how immune development shapes age-dependent infection outcomes, and what balance of vertical, postnatal, and horizontal transmission maintains viruses in nature. Integrating these efforts with ecological studies will be essential for defining the mechanisms sustaining mammarenavirus persistence in reservoirs and spillover risk to humans.

### Hantaviridae

Orthohantavirus research has advanced through studies of four key reservoir systems: Seoul (SEOV)*-Rattus*, Puumala (PUUV)*-Myodes*, Hantaan (HNTV)*-Apodemus*, and Sin Nombre (SNV)*-Peromyscus* (Fig. 1B). Unlike Old World mammarenaviruses, hantavirus infection of rodents elicits strong neutralizing antibody responses thought to preclude vertical transmission. Thus, horizontal transmission—possibly via aggressive behavior—is thought to predominate (134). Despite widespread viral antigen across tissues, infections are typically persistent and subclinical (135). Compared to mammarenavirus models, particular hantavirus-host pairings offer additional insights into immune mechanisms governing persistence, with a skewing of the traditional Th1 antiviral response toward a regulatory T cell response limiting immunopathology (136–138).

#### Seoul virus and *Rattus*

Like *Mus, Rattus* has long served as a key model organism for biomedical and behavioral research. Despite their utility, domesticated rats differ from wild *Rattus*, and knowledge of their ecology in the context of SEOV infection remains limited (71). SEOV, like mammarenaviruses, shows age-dependent pathogenesis in laboratory rats. Neonatal infection results in persistence with SEOV RNA and infectious virus detected up to 6 months (139). However, delaying infection to day 10 of life yields a transient infection cleared within weeks (134, 139, 140). During persistence, SEOV RNA is widespread but most readily detected in the lungs, kidney, and liver alongside vascular smooth muscle and endothelial cells (134, 139, 140). While this generally yields no clinical disease, there is evidence of mild liver inflammation in persistently infected *R. norvegicus* compared to controls (141). Experimental infection of adult rats produces no overt clinical disease, and only a subset remains viremic after several months, consistent with the development of neutralizing antibodies and subsequent viral clearance.

Like mice, domesticated and laboratory rats differ physiologically and immunologically from wild *Rattus* (67, 70), and only two studies, to our knowledge, interrogate infection in more “wild” populations. First, studies of naturally infected feeder rats found viral RNA in lungs, kidney, and liver, and mild hepatitis in seropositive adults, while juveniles in those cohorts rarely carried viral RNA, suggesting inefficient vertical transmission and a protective role for maternal immunity (141, 142). This is further supported by maternal antibodies transmitted either *in utero* or via breastmilk, which can protect neonates from infection (143–145) and suggests the natural transmission route for SEOV is predominantly horizontal. Second, in comparing experimental infection with naturally infected wild *R. norvegicus,* naturally infected rats had no detectable CTL responses but retained an antibody response to infection (146). As with other models, *Rattus* species must balance persistent viral replication with immunity; in general, this is solved by SEOV-mediated increases in immune regulatory responses and decreases in pro-inflammatory responses to prevent immunopathology (137, 147, 148). Reported changes include upregulation of FOXP3 and TGFβ gene expression, expansion of CD4+ CD25+ FOXP3+ regulatory T cells in lungs, and suppression of TNF (137). Viral factors may also contribute to persistence as putative defective viral genomes accumulate during persistency *in vitro* (149). It remains to be determined whether defective viral genomes arise *in vivo,* and, if so, what role they may play in persistency establishment and maintenance.

Sex also strongly shapes viral control and immune activation in laboratory *Rattus* (150, 151). Males tend to have higher SEOV RNA loads, greater shedding, and elevated expression of stress-related genes (150, 152). Females exhibit stronger transcriptional upregulation of immune-related genes (152). This pattern of heightened pro-inflammatory signaling in males and stronger antiviral activation in females continues through adaptive immunity. Males show elevated levels of IFNγ-driven Th1 responses and IgG, while Th2 responses are similar between sexes (150, 151). Parallel findings were seen in a guinea pig model of Lujo mammarenavirus, albeit not in the reservoir (65). Hormonal manipulation alters SEOV RNA levels, with gonadectomy reducing viral RNA in males, while ovariectomy in females or exogenous testosterone in males increases viral RNA (153, 154). This effect on viral replication may be directly mediated by sex steroids, as hormone response elements are present in promoters of key antiviral genes and display higher expression in females (150, 154).

#### Puumala virus and *Myodes*

Experimental infection of adult bank voles (*Myodes glareolus*) with PUUV begins with an acute phase characterized by high viral RNA in saliva, urine, and feces (155–157). This transitions into long-term viremia, with PUUV antigen detectable across lungs, liver, spleen, pancreas, brown fat, small intestine, and salivary glands, for at least 270 days (156, 157). High viral loads in salivary glands and infectious virus in oropharyngeal secretions support horizontal transmission as the primary route (156). Reminiscent of observational immunogenomic studies with *Mastomys* and LASV (158), *Myodes* may exhibit genetic differences in PUUV-endemic versus free regions (159). Experimental infection of *Myodes* from non-endemic areas leads to greater immune responsiveness but lower levels of viral RNA, which may be due to genomic differences in cytokine regulation through TLR and JAK-STAT signaling and impact infection susceptibility (160, 161).

#### Hantaan virus and *Apodemus*

Laboratory models of HNTV infection reveal variable patterns of pathogenesis and transmission, often diverging from field surveillance data (162, 163); however, few studies explicitly examine HNTV dynamics in its natural host, *Apodemus*. In adult *Apodemus* inoculated via the intramuscular route, viremia lasted approximately 1 week and was followed by development of neutralizing antibodies by three weeks post-infection, without apparent clinical symptoms (31, 164). Infectious virus was recovered from the lung, kidney, salivary gland, and liver, and excreted via urine, saliva, and feces for at least 1 year (31, 164). This sustained shedding supports horizontal transmission among cage mates, whereas vertical transmission from females inoculated during gestation was unsuccessful, with *in utero* transmission likely precluded due to the presence of maternal neutralizing antibodies (165). Species-specific differences in immune regulation of HTNV infection have been observed, as murine macrophages exhibit a Notch pathway-mediated “immune brake” to limit immunopathology absent in human macrophages (166).

#### Sin Nombre virus and *Peromyscus*

Among all rodent-borne viruses, SNV in *Peromyscus maniculatus* represents one of the most well-characterized host-viral systems. Following the 1993 outbreak of hantavirus cardiopulmonary syndrome (HCPS) in the United States Four Corners region, an experimental infection model using geographically matched *P. maniculatus* and SNV isolates was established (167). Infection of adult mice yielded a persistent phenotype. During the acute phase of infection (days 5–28), SNV RNA and nucleocapsid (N) antigen were consistently detected across a broad panel of tissues, including lung, heart, brown and white adipose tissues, kidney, liver, and blood (167, 168). During persistency (day 60 and later), mice exhibited either a “widespread” or “restricted” pattern of virus distribution. Widespread mice showed active viremia and broad distribution of genome and antigen across multiple tissues, while in “restricted” mice, the virus was only found in heart, lung, or brown adipose tissues, with the heart being the most consistent depot (167, 169, 170). This pattern was also observed in naturally infected *Peromyscus maniculatus* (170). In restricted animals, there was only evidence of genomic viral RNA, but not antigenomic RNA, suggesting that SNV may induce persistence whereby viral RNA is not actively replicated but maintained in a quiescent manner (169).

Host factors, including age, sex, and geographic origin, significantly modulate SNV dynamics in *Peromyscus*. Females exhibit higher SNV RNA loads in lung and heart tissues relative to males, and juvenile animals display higher viremia than adults (171). Unlike studies with SEOV in *Rattus*, ablation of testosterone levels via castration of adult male *P. maniculatus* had no effect on SNV RNA levels (171). Geographically distinct peromyscine mice experimentally infected with SNV also show varying levels of permissiveness to infection (172, 173). Importantly, there is no evidence of pathology at the tissue level (167–169).

Persistently infected *P. maniculatus* intermittently shed SNV RNA in saliva, with minimal evidence of virus in urine or feces (168, 170, 174). This supports the preferred direct horizontal transmission route, while indirect contact via bedding was not as effective (170, 171). Vertical transmission is not supported by experimental studies and is generally thought to be prevented by maternal antibodies (170, 175). Notably, reproductive success differs significantly between laboratory versus wild *Peromyscus*, as laboratory-bred females produce fewer and smaller litters independent of SNV infection, compared to wild females complicates assessing fitness costs (176). Longitudinal studies in semi-natural enclosure systems indicate that SNV transmission may be influenced by both season and population density. Specifically, seropositive and PCR-positive individuals are more likely to transmit virus to naïve contacts during early summer months or when housed in high-density social groups (177, 178).

SNV induces early downregulation of antiviral gene expression that may promote persistency and minimize immunopathology (167, 168, 171). Inflammatory responses are detectable in the acute phase but are not sustained despite continued viral replication (168, 174). In *ex vivo*-stimulated peromyscine lymphoid tissues, SNV elicits a reduced immune signature compared to heterologous infection with ANDV, a virus normally carried by cricetids (179). At the adaptive level, SNV CD4+ T cell proliferation is limited throughout infection. During the acute phase, CD4+ T cells predominantly express IFNγ, IL4, IL5, and TGFβ1, while persistency is typified by increased FOXP3 expression and co-expression of markers associated with a shift toward a regulatory immune profile that may minimize immunopathology and incapacitate antiviral T cells from clearing infection (174). Neutralizing antibodies are detectable between 7 and 14 days, are at peak between 28 and 60 days, and are sustained through at least 217 days (167, 169). While reservoir-targeted vaccination programs and antivirals have been explored to limit SNV transmission, outcomes are variable (180–182). As with other mechanisms of viral control in wildlife, there may be unforeseen consequences of eliminating SNV in deer mice through the lens of protecting human health.

#### Other orthohantaviruses

Other priority orthohantaviruses remain understudied in their natural hosts but display infection dynamics parallel to those described above. In laboratory-bred *Sigmodon hispidus*, infection with Black Creek Canal virus (BCCV) produces a biphasic pattern with acutely high viral loads, followed by reduced viral RNA expression but sustained shedding of infectious virus (183). A similar persistent pattern occurs with Caño Delgadito virus (CDGV) in *Sigmodon*, marked by continued shedding in oropharyngeal secretions and urine (184). There was no sex bias in susceptibility to infection, and horizontal transmission to naive cage mates was efficient, but unlike studies with SNV and SEOV, offspring of BCCV-infected *Sigmodon* became infected despite the presence of neutralizing maternal antibodies (185). Immune profiling reveals convergent mechanisms underlying persistence across orthohantavirus-host pairs. In ANDV-infected *Oligoryzomys* and Bayou virus (BAYV)*-Oryzomys*, JAK-STAT suppression and increased caspase-1 expression mirror immunoregulation patterns in SEOV and SNV infections (179, 186, 187). BAYV-endemic populations of *Oryzomys* exhibit SNPs in the JAK-STAT, MHC, and NFκB pathways, implying that alterations to host immunogenetics may favor immunotolerance, as is suggested with *Myodes* and *Mastomys* (158, 187).

#### Future directions

Although the foundational systems above have provided important insights into viral persistence and immune regulation, several orthohantavirus-host pairs remain unexplored experimentally, and the existing models would benefit from new reagents and tools to better query host-driven responses. Future work should expand infection systems in authentic orthohantavirus reservoirs and link them with direct studies of naturally infected, wild rodents. Key parameters to focus on include the natural history of infection, the impact of infection on host fitness, general ecological surveillance, and comparative genomics. Critical questions remain regarding which host immune mechanisms enable viral tolerance, how genetic diversity and co-infections shape persistence, the costs and benefits of infection on rodent health and behavior, and what ecological factors drive shedding and spillover risk.

### Other viral families

Both mpox (MPXV) and Omsk hemorrhagic fever virus (OHFV) remain poorly studied in their natural hosts. No studies, to our knowledge, have attempted OHFV infection of muskrats nor in the primary vector, *Ixodes* ticks (188). MPXV is endemic to Central and West African forests, with African rope squirrels (*Funisciurus anerythrus*) considered a likely, although unconfirmed, primary reservoir (189). Only one study has experimentally infected *F. anerythrus*, resulting in moderate-to-severe disease, including ulcerative lesions, respiratory distress, and prolonged viral shedding (2–3 weeks), depending on the inoculation route. Similar outcomes have been observed in other Sciuridae species, like prairie dogs, with disease resembling human smallpox and efficient transmission via fomites and respiratory routes (190, 191). However, most studies prioritize modeling human disease rather than investigating reservoir host dynamics. The high morbidity and mortality in these systems contrast with subclinical infections typical of other rodent-borne virus systems.

## TECHNICAL CHALLENGES WITH NON-STANDARD LABORATORY MODELS

The growing recognition of gaps in our knowledge regarding the ecology of infectious disease has driven parallel efforts in other high-priority viral systems to study pathogens in their natural reservoir hosts rather than traditional laboratory models (192, 193). Work on bat-borne viruses in the *Coronaviridae, Filoviridae,* and *Paramyxoviridae* families has demonstrated that captive colonies, reservoir-derived cell lines, and species-specific reagents can reveal host-specific immune programming and mechanisms of viral persistence that are hidden by surrogate host systems (194–201). These advances are benefiting research in other viral families and have far-reaching implications outside of virology (202, 203). In contrast, investigations of avian influenza viruses in the *Orthomyxoviridae* family still heavily rely on domesticated poultry (204), although wild Anseriformes and Charadriiformes constitute the primary natural reservoirs (205, 206). While nontrivial to establish and study, authentic systems have enabled valuable insights into zoonotic virus pathogenesis, provided a roadmap for implementation in outbreak scenarios, and highlighted the infrastructure challenges inherent to establishing and maintaining such systems (207).

### Establishing wild-caught and non-traditional rodent colonies

While nontraditional and sometimes challenging, the establishment of wild-caught rodent colonies and near-natural infection models have been successfully undertaken in several examples. For example, to establish the first model of SNV infection in wild-caught *P. maniculatus,* an outdoor, open-air quarantine facility was developed in New Mexico, United States, that enabled the safe handling of potentially hantavirus-infected animals outside high-containment laboratories. This facility, which was located deep within a fenced and patrolled National Wildlife Refuge, featured artificial nest box enclosures (cages) that could sustain wild rodents through extreme weather and predator threats while minimizing exposure risks to research staff (208). Notably, the mice and virus used in this facility were trapped in the same geographic region as the facility, eliminating the possibility of introducing a non-endogenous host or virus into the environment. The facility allowed safe quarantine of wild *Peromyscus* to found a wild-caught breeding colony (176), established the first experimental SNV infection model in *Peromyscus* (167, 169, 170), and later supported investigations into the host-specific immune response to SNV (168, 174, 179). Variations of this facility design were successfully used to study the impact of heavy metals on wild *Peromyscus leucopus* in Illinois, United States (209), and Andes virus transmission between wild *Oligoryzomys longicaudatus* in Chile (210). Increasing use of *Peromyscus* as a laboratory model has led to the maintenance of wild-derived, genetically defined stock that has been integral to research in ecology, immunology, genomics, behavior, and disease modeling (211, 212). Similar approaches have been applied in other rodent reservoirs, as colonies of *Mastomys* and *Calomys* derived from wild founders have enabled the experimental study of LASV and MACV in laboratory settings (62, 107, 111, 113). More recently, the development of semi-natural enclosures for laboratory rodents could facilitate the study of viral dynamics and host responses under near-natural conditions (213–215).

However, the design and husbandry surrounding these facilities present additional considerations. Semi-natural or outdoor enclosures must prevent the escape of study rodents and intrusion from non-study organisms, yet still provide the structural complexity to sustain species-appropriate behavior and welfare (216). Housing density, environmental enrichment, diet formulation, and photoperiod management often require refinement from standard laboratory mouse protocols to support these diverse species and may be difficult to maintain in both natural and artificial laboratory environments (217, 218). Establishing colonies from wild founders can also introduce practical challenges, including acclimation to captivity, variation in reproductive success, and eventual domestication-related changes, as well as concerns around appropriate quarantine periods prior to introduction to traditional animal facilities (176, 208, 217). Regulatory layers, including wildlife permits for sourcing and transport, biosafety review, and environmental assessments, add burdens to research staff in implementing these more authentic model systems. Despite the logistical, technical, and biosafety hurdles, it is feasible to establish wild-caught colonies and near-natural infection models. Notably, broad efforts are refining protocols for this work and serve as practical templates for researchers intent on maintaining ecologically relevant reservoir hosts for infectious disease research (214).

### Model characterization

Despite the need to employ non-standard laboratory models for infectious diseases, significant limitations remain in understanding their baseline biology. Even widely used models like the ferret—long recognized as the gold standard for influenza research—were hindered until the annotated ferret genome release in 2014 enabled more sophisticated study of molecular responses to infection (219–221). Likewise, in the study of rodent-borne viruses, we are limited in understanding their natural infection histories due to limited technical expertise, specialized husbandry requirements, poor reagent availability, incomplete genomic annotation, and lack of reference values for genera beyond *Mus* and *Rattus* (222–224). Development of *Mastomys, Calomys,* and *Peromyscus* colonies has expanded the study of these rodents and their infection responses (107, 111, 176, 218, 225); however, research is still limited by a few cross-reacting molecular reagents and poor access to diverse rodent genera in laboratory facilities (138, 226–228). The Zoonomia Project and Vertebrate Genome Project have drastically increased the availability of complete, error-free genomes across mammalian orders that will enable further study into host-specific responses to infection and have already provided key insights into reservoir biology and the conserved basis of human disease (229–231). These efforts will also aid in developing genetic knockout and transgenic models for mechanistic research in diverse rodent species, as has been accomplished for STAT2^-/-^ and hACE2 Syrian hamsters (232, 233).

Model characterization of wild-derived rodent colonies must begin with recognition that their immune systems fundamentally differ from specific pathogen-free laboratory strains (67, 68). Wild *Mus* typically exhibit heightened basal immune activation, expanded memory T cell compartments, elevated immunoglobulin, and transcriptional signatures consistent with chronic exposure to environmental antigens (67, 234). Similar patterns have been reported across diverse taxa*,* highlighting that these immune phenotypes may be broadly relevant (228, 235). In contrast, decades of breeding under barrier conditions have selected laboratory *Mus* for reduced immunological variability, attenuated inflammatory responses, and reproducible but ecologically narrow phenotypes. The “wilding” of laboratory mouse strains through the use of pet store mice, co-housing and microbiota transfers, and semi-natural enclosures partially restores immune maturation, capturing features of memory differentiation, cytokine responsiveness, and microbial interactions that resemble natural populations and have found use in translational work for human health as well (236–241). Incorporating these eco-immunology principles allows experimental infection studies to better reflect authentic host-virus interactions and better bridge the gap between field and lab research, critical for understanding the natural history of zoonotic viruses (242, 243).

### High-containment requirements and restrictions

Most medically relevant rodent-borne viruses require animal biosafety level (A/BSL) 3 or 4 containment. For example, SNV, while a risk group 3 virus, requires ABSL-4 containment for *in vivo* work conducted indoors in the United States. This high containment requirement drives up infrastructure needs, training burdens, operational costs, and regulatory hurdles to keep researchers, laboratory rodents, and the public safe (244). However, it also imposes scientific constraints around enhanced PPE, restricted handling time, and modified housing conditions that could influence experimental outcomes in the natural hosts. As most ABSL-3/4 facilities have inherent limits to capacity and experimental throughput, it constrains sample sizes, replication, and slows progress on fundamental reservoir biology. As outlined earlier, one strategy that successfully reduced the bottleneck regarding infection studies with SNV was the creation of an outdoor, open-air “ABSL-4” laboratory (208). This facility, which cost less than $20,000 in 1998, also allowed researchers to mimic natural ecological conditions while maintaining biocontainment, thereby reducing the disconnect between laboratory studies and real-world host-pathogen interactions.

### Surrogate model systems

Additionally, surrogate model systems not just for the study of human disease but also for natural history studies of reservoirs at the ABSL-2 and -3 levels would reduce the biocontainment bottleneck (58). Maporal hantavirus (MAPV), a New World hantavirus not linked to human disease and principally carried by *Oligoryzomys delicatus,* can model infection responses in the natural *Peromyscus* host at ABSL-3 (245, 246). Similarly, MORV, a close relative of LASV and also carried by the natal multimammate mouse, is not known to infect humans and may have utility as a surrogate host-virus system (133, 247). This system may be especially useful in determining host and viral barriers to infection, as Mobala virus (MOBV), another non-human pathogenic arenavirus carried instead by *Praomys* species, may fail to establish persistency in *M. natalensis* (247–249). These systems would enable scalable organismal experiments that would be impractical at higher containment levels. On the viral side, application of pseudotyped viruses in reservoir hosts could provide an intermediate strategy to preserve receptor usage while avoiding high-containment requirements of the authentic virus, as was heavily implemented with SARS-CoV-2 in A/BSL-2 laboratories (250). This may be especially useful in A/BSL-2 evaluation of entry, host range, and genetic barriers to cross-species transmission. Use of complementary non-pathogenic model systems, such as Pegiviruses and Arteriviruses, may also be key in illuminating conserved mechanisms underlying viral persistence (251, 252).

Still, considerations of species-specific responses to viruses must be made even among closely related rodent species, which, despite their relatedness, can have markedly different receptor repertoires, immune responses, and co-evolution history with regard to closely related viruses (179, 253, 254). Comparative studies between SNV and Andes virus (ANDV) infection of *Peromyscus* (179) suggest that matched virus-reservoir pairs result in lower immune activation than heterologous pairs and could skew the interpretation of results in these surrogate systems. Surrogate arenavirus systems also illustrate similar issues, as the above example of MOBV in *Praomys* fails to establish persistence in *M. natalensis* despite its close relation to MORV, which underscores the specificity of reservoir-virus compatibility (247–249). This has also been observed with closely related Cricetids and JUNV, as viral persistency apparently fails to establish in *C. laucha* but can in *C. musculinus* and *A. molinae* (60, 114, 116, 117). Taken together, implementation of organismal surrogate systems and support from strategic use of pseudotyped virus work in authentic reservoirs can accelerate discovery while reserving A/BSL-3 and -4 capacity for validating mechanisms in matched virus-reservoir pairs.

### Viral genetics and susceptibility

It is essential to consider not only the host’s response to infection but also viral isolate variability. Viral strains can exhibit different dynamics depending on isolation history and laboratory adaptation (255, 256). For example, emerging influenza virus strains may not readily isolate from animal hosts when using standard MDCK cells (257). Use of a matched host-viral species system, like isolating and expanding PUUV in *Myodes*-derived cells, may minimize genetic divergence from the parental sample (258, 259). This reduces the risk of laboratory-induced adaptations that may skew downstream infection dynamics and preserves tissue tropism and immune modulation seen during natural infection. However, establishing these cell culture systems is extremely difficult with prohibitive costs and training barriers to broad implementation. Similarly, use of laboratory-adapted viral strains, like LCMV WE or Clone 13, may not recapitulate host-virus dynamics seen in nature compared to a more “wild” isolate and should be considered when assessing host responses to infection, as experimental studies with JUNV*-Calomys* and HTNV*-Rattus* illustrate (260–263). Geographically matched host and virus can also dictate study outcomes and are an underrecognized component of linking pathogenesis and immunology studies to disease ecology (172). However, successful isolation of “wild” viruses can be time-consuming and often met with low success rates (167, 264, 265). Ultimately, employing systems that closely replicate natural host-virus interactions is crucial for better understanding viral pathogenesis and improving experimental accuracy.

## CONCLUSION

**Fig 2:**
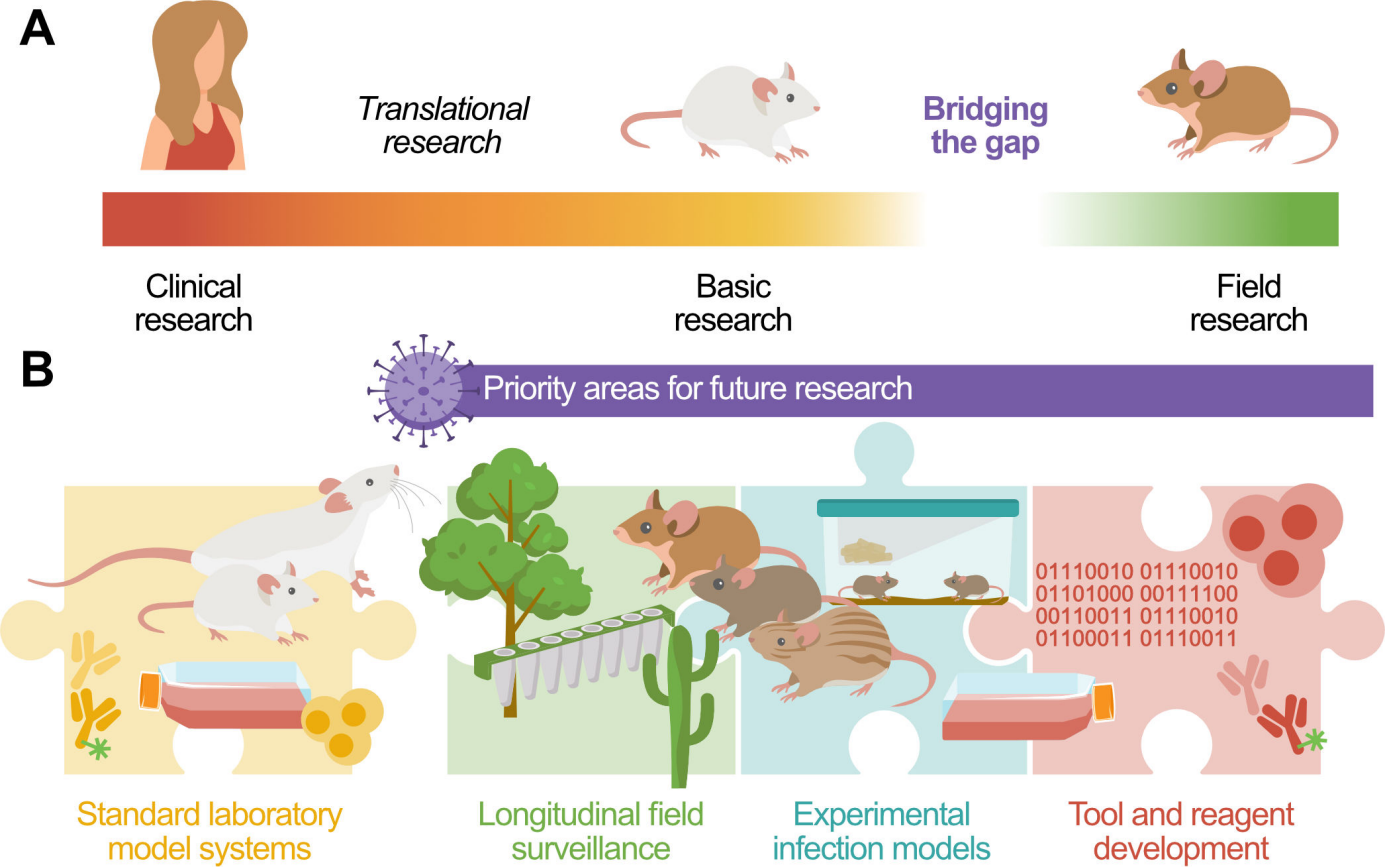
Proposed integration of surveillance, experimental infection, and laboratory studies for pandemic preparedness. (**A**) Current reliance on standard laboratory model systems for the study of zoonotic viruses leaves significant gaps in our understanding of their true biology, and progress should mirror the push for more translatable science in pre-clinical work. (**B**) To address these deficiencies, it is essential to supplement traditional field surveillance with more robust and well-characterized experimental infection models. Such models, in tandem with the development of cellular, molecular, immunological, and bioinformatics toolkits, will be pivotal for enhancing pandemic preparedness and enabling more accurate risk assessments. Artistic representations were designed by Freepik (rodent species, tree, virus, and puzzle pieces), studiogstock (human) or adapted from the Servier Medical ART resource (laboratory equipment) and are used under a CC BY 4.0 license or were designed by the authors.

Rodent-borne zoonotic viruses pose a significant global public health threat (266, 267). Their widespread distribution, coupled with increasing human encroachment and climate change, has heightened outbreak risks (4, 268). Studying these viruses in both laboratory and natural contexts is essential (66, 269). Laboratory studies allow controlled investigation of viral kinetics and pathogenic effects of viral mutations or host factors, while observational field studies offer crucial insights into real-world transmission patterns, host-virus co-evolution, and ecological drivers of emergence. Developing standardized experimental infection models in authentic reservoir species can illuminate key differences underlying viral pathogenesis in rodents and humans. For example, SNV induces a strong regulatory T response in *Peromyscus,* but a comparatively weak response in patients with hantavirus cardiopulmonary syndrome (174). Similarly, experimental spillover studies across divergent *Mus* and *Peromyscus* rodent lineages demonstrate that even when cross-species infection is possible, transmission efficiency may remain limited and viral genomes experience strong purifying selection, highlighting the evolutionary constraints shaping host switching (253). Integrating the experimental control of laboratory systems with the authenticity of field studies will improve our ability to develop effective prevention and control strategies and support the development of vaccines and other therapeutics for these high-priority pathogens (270). In this sense, these experimental model systems represent a necessary extension of the translational paradigm toward one that not only connects bench to bedside but also addresses the relative lack of bridging between controlled laboratory science with real-world field ecology (Fig. 2A).

Despite their importance, laboratory-based infection models have inherent limitations. Wild rodents are neither sterile nor specific pathogen-free, and co-infections modulate host immunity and survival (271–275). Behavioral changes in rodents may alter transmission dynamics as in the classic example of *Toxoplasma* in rodents (276) and other pathogen-driven shifts in activity or aggression that promote viral spread (128, 178, 277, 278). These challenges illustrate the difficulty of replicating natural ecological complexity within the artificial laboratory and underscore the wealth of information field studies provide (177, 278–280). This is exemplified by the need for geographically matched rodent and viral strains complicating the first SNV*-Peromyscus* model and concerning MACV and LATV pathogenesis in *Calomys* (116, 167). At the same time, expanding the molecular toolkit for wild populations allows aspects of viral dynamics and host responses to be measured directly in wild populations and incorporated into laboratory experiments (173, 281–283). Use of modernized museum collections that utilize a holistic collections strategy, indexed biorepositories, and extended voucher specimens will also be invaluable as a tool for the cross-disciplinary study of zoonotic viruses (284). Framing this work within a One Health perspective may offer a more comprehensive strategy for studying the natural history of zoonotic viruses and highlights how complementary field and laboratory approaches are essential to predict and mitigate emerging infectious diseases (Fig. 2B).

Rodents remain indispensable in biomedical research; however, significant gaps remain in our understanding of their biology when viewed through the lens of their wild existence and interactions with viruses in nature (67, 285, 286). The laboratory rodent immune system is well-characterized; however, we know little of the wild rodent immune system (67–70). Studies using “dirty” mice have illuminated how rodents interact with pathogens in ways more closely mimicking their wild relatives and have provided insight on the evolutionary constraints during spillover across the species barrier (238, 253). The use of outbred strains better reflects the genetic diversity and immune variation found in natural rodent populations (287, 288). This has improved our understanding of rodent biology and increased translatability to human disease. To fully grasp the natural history of rodent-borne viruses, it is essential to complement this with research in wild rodents, whose interactions with pathogens are shaped by ecological and evolutionary pressures standard laboratory models cannot replicate. Bridging the gap between controlled laboratory models and the true biology of reservoirs in nature is essential for advancing our understanding of zoonotic viruses and improving our pandemic resilience.
